# Calcified Amorphous Tumor of the Heart with Purple Digit

**DOI:** 10.15171/jcvtr.2014.023

**Published:** 2014-12-30

**Authors:** Feridoun Sabzi, Hosein Karim, Babak Eizadi, Reza Faraji, Nasrin Javid

**Affiliations:** ^1^Department of Cardiovascular Surgery, Imam Ali Heart Center, Kermanshah University of Medical Sciences, Kermanshah, Iran; ^2^Department of Pathology, School of Medical, Kermanshah University of Medical Sciences, Kermanshah, Iran; ^3^Yazd Cardiovascular Research Center, Shahid Sadoughi University of Medical Sciences, Yazd, Iran

**Keywords:** Tumor, Heart Neoplasms, Right Atrium

## Abstract

A calcified amorphous tumor (CAT) of the right atrium (RA) is an exceedingly rare non-neoplastic cardiac mass. It was initially described in 1997 and only a handful of cases has been published so far. We present a case of tumor in 77-year-old male, in the RA that attached to the rim of the fossa ovalis, with classic pathological and clinical findings. Under cardiopulmonary bypass (CPB) and bicaval and aortic cannulation and cardioplegic arrest, right atrial mass, was resected and septal defect was repaired with a fresh pericardial patch. Pathological exam of the mass revealed CAT. The patient had an uneventful hospitalization and his blue discoloration of finger recovered normally.

## Introduction

Calcified amorphous tumor (CAT) is placed in the category of calcified right atrial mass. CAT defined histologically as a cellular matrix with an amorphous fibrin strand with no viable cell. The only exception is myxoma because in necrotic myxoma, some myxoma cell may be found. The following condition must be ruled out before engraving name of CAT in calcified cardiac mass. These conditions consist of chronic renal failure, metabolic abnormalities in serum calcium, hyperparathyroidemia, disturbances in vitamin D_3_ metabolism, hyperuricemia, inherited or acquired thrombophilias or antiphospholipid antibody syndrome.^1^ Right atrial mass of the heart consists of a cardiac mass with different nature that causes symptoms due to mechanical obstruction of tricuspid valve, embolization of tumor or calcified fragments to the pulmonary system or constitutional symptom of tumor. The presence of right atrial mass in opposed to others cardiac tumors was affected by co-moribund disease of patient such as hemodialysis or hepatic failure and tophaceous gout. During differential diagnosis of right atrial mass, cardiac neoplasia, especially myxomas, fibromas, lipomata and papillary fibroelastoma must be considered, particularly if they are calcified and so are conditions involving infection, thrombosis or tuberculoma, histoplasma, calcified hydatid cyst and calcified amorphous tumor.^2^ Cianciulli revealed that however others right atrial mass such as HIV infection, viral or bacterial vegetation, thrombophilia, tumor, and thrombus are rare but may be associated with serious complications including pulmonary embolism.^3^ Kronik found that in situ thrombosis is rare in a structurally normal heart and usually can occur in thrombophilia, central vein pressure line in right atrium (RA), in some particular type of malignant tumors, low cardiac output states, cardiomyopathies, atrial fibrillation, renal failure and some systemic diseases.^4^ Our patient’s thrombus was not mobile or free floating. CAT of the heart mimics malignancy or causes symptoms due to obstruction or embolization of calcify fragments. Calcified cardiac tuberculoma are infrequent and there is usually a history of tuberculosis.

## Case report

**Figure 1:**
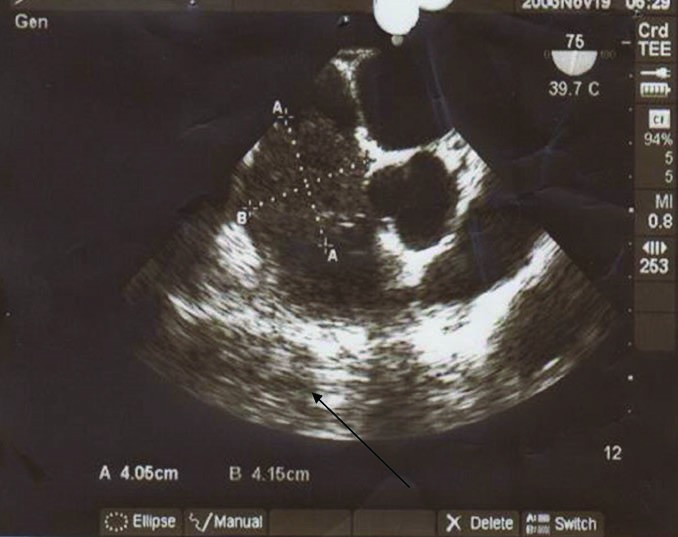
Trans esophageal echocardiography (TEE) shows huge mass in right atrium.

**Figure 2:**
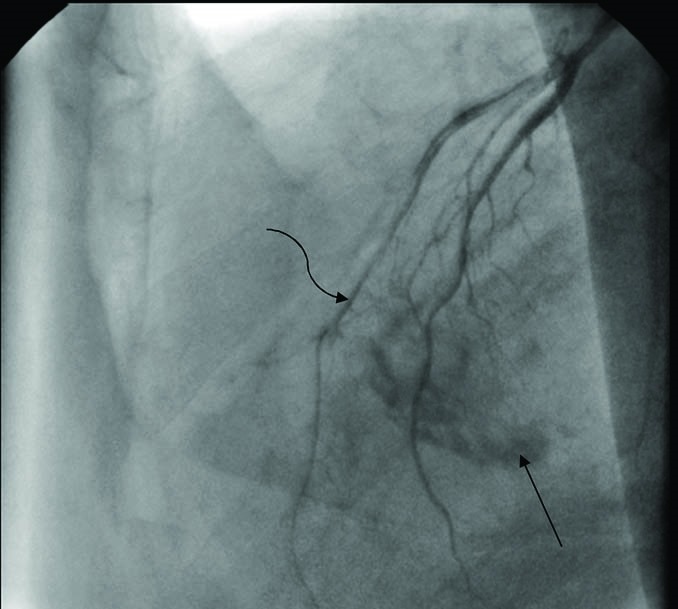
Coronary angiography shows shadow of calcification of tumor.

**Figure 3:**
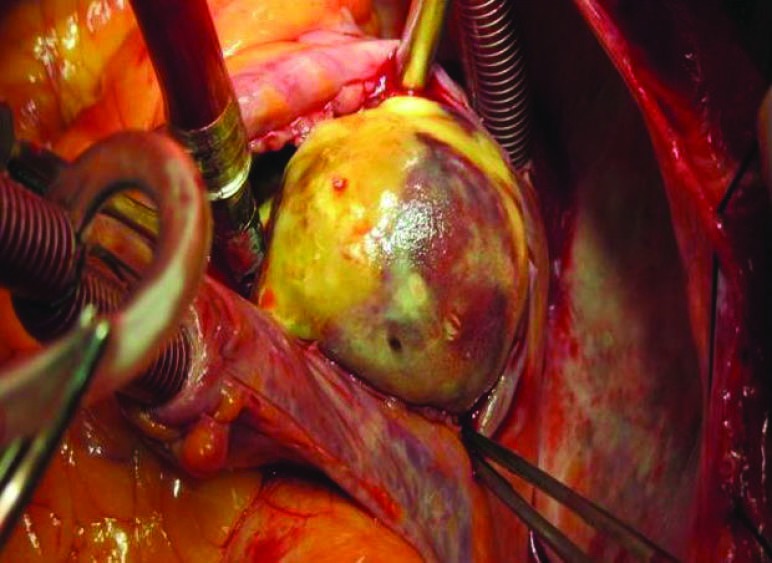
Shows intraoperative right atrial mass.

**Figure 4:**
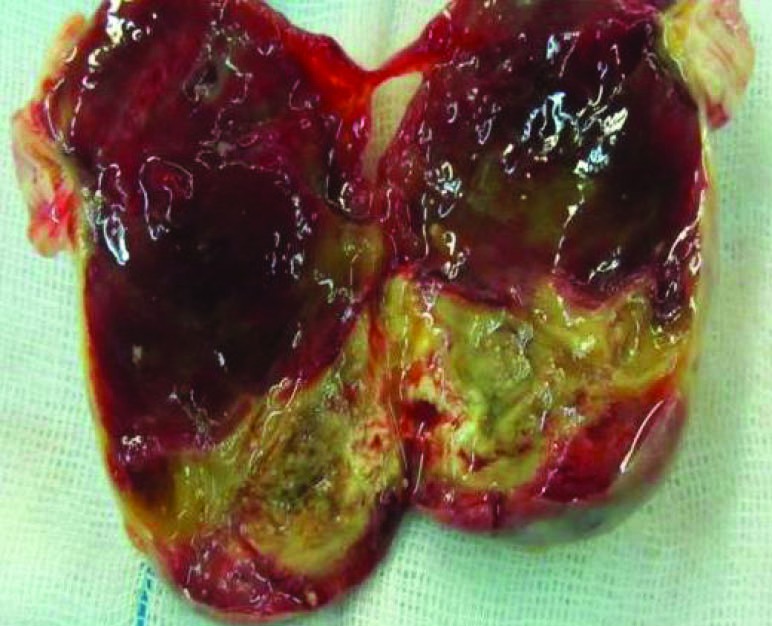
Shows calcification in basis of tumor.

**Figure 5:**
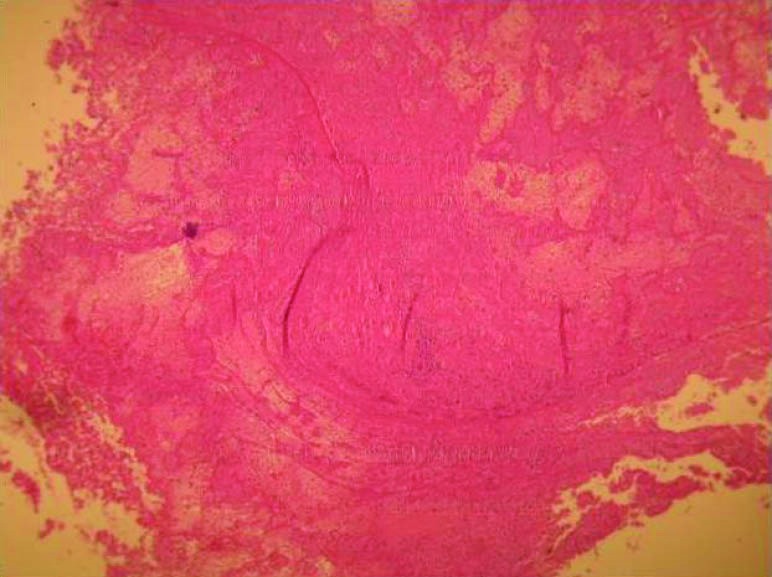
Photomicrograph showing amorphous eosinophilic fibrinous material along with dense calcification (hematoxylin and eosin (H&E) ×100).

A 77-year-old man presented with progressive dyspnea, fatigue and cough and purple digit. He was diagnosed as having a calcified right atrial mass. Routine laboratory investigations were within normal limits. Complete blood count and blood biochemistry were within normal limits, except for prolonged erythrocyte sedimentation. The protein C level was 78% (normal: >70) and protein S level was 80% (normal: 65%).There are no abnormal values of antithrombin III, lupus anticoagulant, and anticardiolipin antibodies, HLA-B5, HLA-B27, anti-nuclear antibody and rheumatoid factor were negative. Electrocardiogram was normal and chest radiograph showed a calcified shadow retrosternally. Tran esophageal echocardiography showed a right atrial mass measuring 4 × 4 cm with calcification but small fossa ovals defect that was masked by tumor was not reported (Figure 1). Angiography revealed normal coronary artery with mobile calcified mass in RA that feeds by a small collateral artery (Figure 2). A clinical possibility of calcified right atrial myxoma was considered. A physical exam revealed cool and cyanotic left fingers. Our patient underwent cardiac exploration and removal of the mass. Intra-operatively, a calcified mass measuring 4 × 4 cm was noted in the RA with a single site of attachment to fossa ovalis septum (Figure 3). The mass was well-circumscribed with massive calcification more in the basal attaching site (Figure 4). The entire tissue was processed for histopathology. Sections showed a lesion composed of a background of eosinophilic amorphous material, possibly degenerated fibrin, with areas of dense calcification and focal chronic inflammation (Figure 5). No myxomatous or lipomatous or fibromatous tissue was seen and a final diagnosis of CAT of the heart was rendered. The patient was started on aspirin, pentoxifylline and heparin. The cyanotic left hands were kept warm and undue handling was avoided. In 5^th^ operating days, extremity became warm with normal texture and colors.

## Discussion

Reynolds for the first time described the CAT of the heart as a very rare non-tumoral and intracavitary mass in 1997.^5^ Since then, 17 cases of cardiac CAT have been reported in the medical literature right atrial calcified mass are rare and the most common predisposing factor, are old thrombosis.^6^ Despite different pathological nature of right atrial calcified mass, excision of the lesion is important due to the potential danger of obstruction or embolization and also for accurate diagnosis and therapy. The clinical presentation of cardiac CAT is similar to that of other cardiac masses, i.e. dyspnea, syncope or symptoms related to embolism. Hence, the most frequent clinical impression is a cardiac myxoma, as in our patient. However other differential diagnoses include primary atrial thrombi, lower extremity emboli lodged in the right atrium, inferior vena cava thrombosis with extension to the right atrium, renal tumors with involvement of IVC and RA, nephrotic syndrome with thrombosis of renal vein and emboli to RA and other benign or malignant cardiac tumors.^7^

Tazelaar et al. showed that cardiac fibroma may also be calcified; however, they are located predominantly in left ventricular muscle.^8^ Other causes of calcification in the heart include alport syndrome in patients with chronic renal failure. Some type of myxoma may calcified; hence, adequate tissue processing and sampling is essential to exclude underlying myxoma.^9^ Non endocarditis vegetation’s due to auto immune disease are intimately associated with valve leaflets and may rarely calcified. Calcified echinococcosis can be diagnosed by the identification of the cyst wall and presences of scolices.^10^ Thrombus contains red and white blood cells that with platelet deposited in successive manner and created a laminar shape in thrombus. Blood proteins and phospholipids in these complex, with absorption of calcium ions combined by phosphate ions (PO_4_-) leading to the formation of calcium phosphate complexes and intra thrombus calcification. Predisposing factors for thrombus formation were not observed in some patients with CAT. These factors include, thrombophilia, prolonged bed rest differentiates it from thrombosis clinically. Pathological differential diagnosis of CAT from an organized thrombus include, lack of characteristic laminations of an organizing thrombus and rare presence of hemosiderin.^11^ The contributing factors in pathogenesis of cardiac CAT are not well-known. There are two hypotheses about nidus formation in cardiac CAT. Most authors supposed this hypothesis that intra mural thrombosis can be organized and lead to CAT. Others author support this hypothesis that cardiac CAT is merely an organized and calcified myxoma.^12,13^ This is a hypothesis that supported by the absence of predisposing factors to thrombus formation in some patients. Clinically CAT is very rare in cardiac chamber and these patients have not congestive heart failure, right atrial wall infarction or cardiomyopathy or permanent right atrial endocardial pacemaker. Sousa et al. showed that with absence of pre-mentioned variables, in CAT of the right atrium, thrombotic nidus may not be the only contributing factor in thrombus formation.^10^ The majority of surgically treated CAT were benign mass and cured by surgery.^12,13^

Fealey et al. for the first time, reported a recurrent case of right atrial CAT in a young patient complicated by thromboemboli.^14^ Spencer et al. described a patient with a heavily calcified right atrial thrombus caused by an indwelling central venous catheter and long-term intravenous phosphate infusion.^15^ Mujanovic et al. found that indwelling foreign body may become the source of a life-threatening thrombotic mass, if not recognized and treated appropriately.^16^ In Lewin, case report, a right ventricular CAT caused massive thromboemboli, with fatal outcome.^12^ Hence, these patients need to be kept on follow-up after surgical excision with repeat imaging studies in cases with incomplete resection. Mikhail et al. showed that their case is the first demonstration of completely infracted, right atrial myxoma with only a few clusters of viable cells identified histologically.^17^ Tarantino et al. reported a 5-year-old boy with short-bowel syndrome who receives home parenteral nutrition developed a calcified thrombus that involved the inferior vena cava (IVC) and the right atrium. Symptoms included 3 to 4 months of intermittent fever and 2 months of vague chest pain. Blood could not be aspirated from the IVC catheter and an IVC contrast study demonstrated the calcified thrombus.^18^ Greaney et al. presented a 2 cm mobile mass in the left ventricular outflow tract and symptoms of left heart failure and stroke. Pathological examination revealed it as an amorphous tumor.^19^

## Conclusion

CAT is an exceedingly rare cardiac lesion with an excellent outcome following complete surgical removal. Since clinico-radiologic differentiation from other cardiac masses is not possible in most cases, histopathological examination is the only modality for diagnosis. Hence, histopathologists should be aware of this rare entity in the differential diagnoses of cardiac mass.

## Ethical issues

The study was approval by the Local Ethics Committee.

## Competing interests

Authors declare no conflict of interests in this study.
